# Comparison between Shear Bond Strength of Er:YAG and Er,Cr:YSGG Lasers-Assisted Dentinal Adhesion of Self-Adhering Resin Composite: An Ex Vivo Study

**DOI:** 10.3390/dj8030066

**Published:** 2020-07-01

**Authors:** Paul Nahas, Samir Nammour, Elie Gerges, Toni Zeinoun

**Affiliations:** 1 Department of Restorative and Esthetic Dentistry, Faculty of Dental Medicine, Lebanese University, Beirut 27798, Lebanon; 2 Department of Dental Science, Faculty of Medicine, University of Liege, 4020 Liege, Belgium; S.Namour@ulg.ac.be; 3 Department of Prosthodontics, Faculty of Dental Medicine, Lebanese University, Beirut 27798, Lebanon; eliegerges@ul.edu.lb; 4 Department of Oral and Maxillo-Facial Surgery, Dean of Faculty of Dental Medicine, Lebanese University, Beirut 27798, Lebanon; toni.zeinoun@ul.edu.lb

**Keywords:** dentin, bonding, bond strength, laser etching, self-adhering composite

## Abstract

(1) Background: Bonding composite to tooth structure is still evolving with a substitute for phosphoric acid being the main challenge. Lately, a self-adhering composite (SAC) was developed, promising to simplify bonding to tooth structure. Unfortunately, retention especially to dentin, was not as good as the gold standard three steps bonding system. During the last 2 decades, lasers were used to enhance shear bond strength of composite to tooth structure. However, the literature provided limited information regarding laser efficiency in the immediate, as well as the long term, adhesion success of SACs to dentin. The purpose of our study was to define the optimal irradiation conditions to improve the adhesion of self-adhering flowable resin composite to dentin exposed to Er:YAG and Er,Cr:YSGG laser irradiation. (2) Methods: Seventy-two freshly extracted human third molars, prepared to have flat dentinal surfaces, were randomly divided into three groups (n = 24) including a control group (Group 1) in which dentin was left without laser irradiation. The other two groups (Group 2 and 3) received standardized irradiation at a speed of 1 mm/second with Er:YAG (60 mJ; SSP mode = 50 μs; 10 Hz; fluency of 9.4 J/cm^2^; beam diameter: 0.9 mm; air 6 mL/min; and water 4 mL/min), and Er,Cr:YSGG: 1.5 W; fluency of 17.8 J/cm^2^; turbo handpiece with MX5 short insert; 20 Hz under air/water spray (65% air, 55% water). Self-adhering flowable resin was applied to dentin in all groups. Half of the specimens were stored in water for 24 h while the other half underwent 3000 thermal cycles. Later, all specimens received a shear bond strength test. Fracture observation was done first under a stereomicroscope then by using a scanning electron microscope. (3) Results: The mean values of shear bond strength for both laser-treated dentin groups (Er:YAG laser: 13.10 ± 1.291, and Er,Cr:YSGG: 14.04 ± 5.233) were higher than in the control group 1 (8.355 ± 2.297) before thermocycling. After thermocycling, shear bond strength decreased in all groups as follows: 10.03 ± 1.503, 10.53 ± 2.631, and 02.75 ± 1.583 for Er:YAG, Er,Cr:YSGG, and nonirradiated dentin, respectively. Shear bond strength values showed a significant difference between the control group (Group 1) and both lasers groups (Group 2 and 3). Statistical analysis of stereomicroscope observation revealed no significant difference between laser irradiation and failure mode (*p* < 0.136). SEM observation of the dentin surface in both laser-irradiated groups showed opened tubules, absence of smear layer as well as an increase of resin infiltration into dentinal tubules. (4) Conclusion: Er:YAG and Er,Cr:YSGG lasers enhance self-adhering flowable resin shear bond strength values and improve its longevity by eliminating the smear layer, opening dentinal tubules and increasing resin infiltration into the microstructure.

## 1. Introduction

To date, the three steps bond to tooth structure remains the gold standard bonding method [1]. However, patients’ preferences for esthetic, rather than metallic, restorations and dentists’ requirement for quick, simple and easy handling materials, have led companies to market a new type of one-step bonding system known as the self-etch adhesive system (SEA). Bonding enhancement was the key to simplifying restorative techniques. More progress in the field of resins resulted in the development of self-adhering resin composite (SAC), which combines both bonding and resin into one material. SAC needs no prior application of any kind of acid or bonding, since it has all components embedded into one material. Studies conducted during the past years have shown that SACs could be helpful in pediatric dentistry [2,3,4,5].

Short term results have been reported regarding bond strength of adhesives and self-adhering composite to Erbium laser-irradiated dentin. Koliniotou-Koumpia et al. demonstrated that cavities prepared with lasers are less receptive to adhesive procedures than conventional bur-cut cavities [6]. Moreover, morphological changes in dentin, as well as thermomechanical alterations within resin-dentin interfaces caused by Er,Cr:YSGG laser irradiation, have negatively influenced microtensile bond strength of adhesive systems [7]. Also, higher bond strength was recorded when Vertise Flow was applied with acid etching than with Er:YAG lasers [8].

Until today, literature has provided limited information on the durability of bonding to dentin [9]. Thermocycling is widely used to simulate the oral conditions affecting the bonding of composite to enamel and dentin with time. Extreme temperatures are known to weaken the physicochemical properties of bonding [9,10,11]. There is an absence in published reports related to the effect of thermocycling on bonding SACs to laser-irradiated dentin. Such lack of clear information, and difficulty to standardize laser parameters and irradiation techniques, as well as the longevity of bonding of SAC to dentin, would jeopardize long term clinical results and lead practitioners into doubting the use of Er:YAG and Er,Cr:YSGG lasers as a prior treatment for bonding SACs to dentin. 

Unfortunately, many studies reported that SAC’s performance was not comparable to standard three or two steps etch and bond adhesive systems whether it is used on enamel, dentin or even in the case of direct composite repair [12,13,14,15]. Although SACs exhibited lower shear bond strength than one step self-etch bonding systems, previous studies assessing marginal sealing reported a comparable performance [15,16]. Moreover, improving bonding of SAC to dental hard tissues was only successful when phosphoric acid was used [17]. Consequently, modifying the dentin surface could enhance shear bond strength (SBS) values of SACs. This may be explained by the need to eliminate the smear layer in order to facilitate the SAC’s resin interaction with dentinal structure [18]. Hence, smear layer removal can be performed by the use of Erbium lasers such as Er:YAG (2.94 μm) and Er,Cr:YSGG (2.780 μm) at low level energy density [19,20,21]. It was interesting to verify the ability of Erbium lasers to enhance the shear bond strength of SACs. On the other hand, a previous study reported the efficiency of low level energy (60 mJ) of an Er:YAG laser on SBS values of SACs when compared to nonirradiated dentin [20].

The purpose of our preliminary ex vivo study was to check if Er:YAG and Er,Cr:YSGG lasers could improve the immediate bond strength of self-adhering resin composite to dentin as well as improving its longevity when tested under extreme heat and cold conditions.

The null hypothesis supposed that laser irradiations would not improve the bond strength of SAC to the prepared dentin.

## 2. Materials and Methods 

### 2.1. Teeth Collection and Storage

Approval was obtained from the Lebanese University Ethics Committee, Number: CUMEB/D115/42018(approval date 28/2/2018). Seventy-two freshly extracted human third molars were collected. The extraction of teeth was done for unknown reasons and independently to our study. All teeth were free from any restoration, fracture, cavities, caries or other pathology. Teeth were immediately stored in 1% thymol solution for disinfection for one week at room temperature and then washed abundantly with tap water for 2 h to eliminate thymol residues.

In our study we had 3 groups of 24 specimens each (control group, Er:YAG group and Er,Cr:YSGG group). The total number of samples was 72 teeth. The confidence level was set to 95% (z-score = 1.96). The margin of error was 11.55% and the population proportion was 50%.

### 2.2. Specimen Preparation

**Figure 1:**
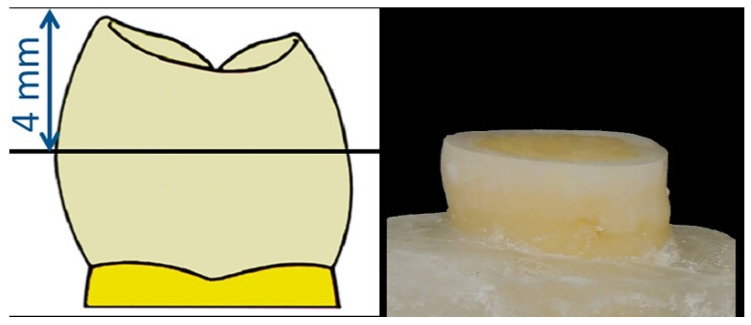
Crowns cut parallel at 4 mm from the occlusal plan.

All specimen preparations were performed by the same operator. Teeth were cleaned with an ultrasonic scaler and fixed 2 mm below the enamel–cementum junction into a resin acrylic (Paladur, Heraeus Kulzer, Inc., South Bend, IN, USA). Crowns were cut parallel to the occlusal plan at a distance of 4 mm from the highest cusp (Figure 1) using a slow (250 rpm) rotating diamond blade (IsoMet 2000, Buehler, Ltd., Lake Bluff, IL, USA). All dentinal flat surfaces were standardized using wet-grounding with 320 then 600 grit silicon carbide papers (Matador, Wasserfasten, Germany). Finally, a stereomicroscope (Leica/Meyer Instruments, Houston, TX, USA) with 20× magnification was used to insure that no pulp was exposed and that no remnants of enamel were visible except at the periphery of the prepared specimens.

All teeth were randomly assigned to three groups (n = 24). The first group did not receive any laser irradiations and served as the control group (Group 1). The other two groups (Group 2 and 3) received laser irradiation as recommended by the manufacturer. Group 2 received Er:YAG laser wavelength 2.940 nm (Fidelis; Fotona, Medical Laser, Ljubljana, Slovenia), 60 mJ of pulsed energy and a fluency of 9.4 J/cm^2^ in a noncontact mode using handpiece H02 with Super-Short Pulse mode (SSP, pulse duration: 50 µs), frequency of 10 Hz under an air/water spray (air, 6 mL/min, and water, 4 mL/min) and 0.9 mm as the beam diameter at the impact point. Group 3 received Er,Cr:YSGG laser wavelength 2.780 nm (Water Lase MD System, BioLase Technology Inc., San Clemente, CA, USA), 1.5 W with an energy per pulse of 35 mJ and an energy density of 17.8 J/cm^2^. The energy was chosen based on a previous study in a noncontact mode using a turbo handpiece with an MX5 short insert with a diameter of 0.5 mm at 20 Hz frequency under an air/water spray (65% air, 55% water) [22]. Delivered energies were verified and calibrated with a power meter (UP19K-15S, Gentec-EO, Québec, QC, Canada). Laser irradiation was performed at a speed of 1 mm/s in a custom-made 2D computer numeric control machine (CNC), standardizing dentin irradiation and eliminating any possible human operator error. 

SAC was applied on all samples (Vertise Flow, Kerr, Orange, CA, USA), by rubbing the flat dentinal surface for 20 s using a branded microbrush. All materials were light cured for 20 s (Demi Plus LED Light Curing System, Kerr, Orange, CA, USA) followed by a 2.38 mm × 2 mm cylinder build-up of flowable composite in a prefabricated mold insert from Ultradent (Ultradent Products, Inc., South Jordan, UT, USA). For all specimens, the light curing tip was 1 mm from the top of each mold. 

In each group, only half of the specimens (n = 12) were subjected to 3000 thermal cycles (MPC, ElQuip, São Carlos, SP, Brazil). Alternation was done between 5 °C and 55 °C with a 5 s transfer time and a 30 s dwell time to simulate a short-term equivalent to 3 months of hydrothermal stress conditions. The other half of the specimens (n = 12) were stored in water for 24 h.

### 2.3. Mechanical Shear Stress

A Shear bond strength (SBS) test was applied on all samples using a notched edge in a shear bond strength testing machine (Ultradent Products, Inc., South Jordan, UT, USA) at a crosshead speed of 1.0 mm/min. SBS results were registered in megapascals (MPa). Fractured areas were observed using a stereomicroscope (Leica/Meyer Instruments, Houston, TX, USA) at 20× magnification in order to assess failure mode according to the Osby et al. modified adhesive remnant index (ARI) as follows: [23]

Score 1: All of the adhesives remained on the tooth.
Score 2: More than 90% of the adhesives remained on the tooth.
Score 3: 10–90% of the adhesives remained on the tooth.
Score 4: Less than 10% of the adhesives remained on the tooth.
Score 5: No adhesive remained on the tooth.

ARI was used because SACs have their bonding integrated into the composite. 

All Specimens were cut longitudinally in the middle of the bonded area and perpendicularly to the irradiated dentin, creating two surfaces for scanning electron microscope (SEM) observation (occlusal and lateral view). Later, all surfaces were dehydrated in a graded series of aqueous ethanol (25, 50, 75, 90 and 100%) with a 20 s treatment for the first three ethanol concentrations and then 30 s for the 90% and one hour for the last ethanol concentration (100%). Dentin surfaces were gold-sputtered and observed for topographical changes under SEM at 3000× magnification.

### 2.4. Statistical Analysis

All groups passed the normality assumptions of Shapiro-Wilk *p* < 0.05. A two-way ANOVA was used to analyze the effect of laser irradiation and aging on SBS of SAC, followed by Newman-Keuls adjusted multiple comparisons test to determine differences between groups. The effect of laser irradiation and aging on the failure mode of the bonded composite was tested separately using Pearson’s Chi-Square Test. Statistical analysis was done using GraphPad Prism (GraphPad Software, Inc., San Diego, CA, USA). *p* value ˂ 0.05 was considered statistically significant.

## 3. Results

### 3.1. Shear Bond Strength Test

Descriptive statistics for the six groups showed an increase in the mean shear bond strength of SAC from 8.355 ± 2.297 MPa for Group 1 (control group) to 13.10 ± 1.291 MPa and 14.04 ± 5.233 MPa, respectively, for Group 2 and 3 (laser-irradiated dentin) after 24 h of water storage. After thermocycling, mean SBS values decreased in all groups. Group 1 exhibited a dramatic drop to 2.75 ± 1.583 MPa. In Groups 2 and 3, mean SBS decreased to 10.03 ± 1.503 MPa and 10.53 ± 2.631 MPa, respectively, but kept a mean value comparable to and even better than Group 1 (2.75 ± 1.583 MPa) (Table 1).

A Newman-Keuls multiple comparison test for samples that were only stored in water (WS) exhibited significant differences between the nonlasered group (WS-Group 1) and both laser irradiated groups (WS-Group 2 and WS-Group 3), while WS-Group 1 showed no significant difference compared to laser irradiated groups after thermocycling (TC-Group 2 and TC-Group 3). Er:YAG and Er,Cr:YSGG groups that were only stored in water (WS-Group 2 and WS-Group 3) showed no significant differences between them (Table 2). 

When observing thermally cycled groups (TC), there was a significant difference between the nonlasered group (TC-Group 1) and both laser irradiated groups (TC-Er:YAG and TC-Er,Cr:YSGG), while laser-irradiated groups revealed no significant difference between them (TC-Er:YAG and TC-Er,Cr:YSGG). 

### 3.2. Stereomicroscopic Analysis

Failure modes observed with a stereomicroscope at 20× magnification did not show any record for score 1 and 2 in all observed groups. It is clear that before thermocycling, score 3 (10–90% of the adhesives remain on dentin), score 4 (less than 10% of the adhesives remain on dentin) and score 5 (no adhesives left on dentin) dominated all groups. Samples in control group 1 that were stored in water (WS-Group 1) recorded a percentage of failure of 12.16%, 33.34% and 54.5%, respectively, for score 3, 4, and 5. In laser-irradiated dentin, the percentage of failure had a minor change with, respectively, 32.19%, 41.66% and 26.15% for WS-Group 2, and 33.84%, 40.06% and 26.10% for WS-Group 3, within score 3, 4, and 5.

After thermocycling, a remarkable rise occurred to the percentage of failure in the nonirradiated group (TC-Group 1). Only 2 samples had a score of 4, and the rest (10 samples) fell into score 5. In contrast, laser-irradiated groups (TC-Group 2 and TC-Group 3) maintained higher percentages with, respectively, 25.66% and 24.96% in score 3, 32.34% and 31.34% in score 4, and 42% and 43.70% in score 5 (Table 2).

Additionally, fracture mode was analyzed using a Chi-Square test separately for laser-irradiation and thermocycling procedures. Both variables showed no statistically significant differences with *p* = 0.136 for laser-irradiation and *p* = 0.091 for the thermocycling procedure.

### 3.3. SEM Topographical Observation

**Figure 2:**
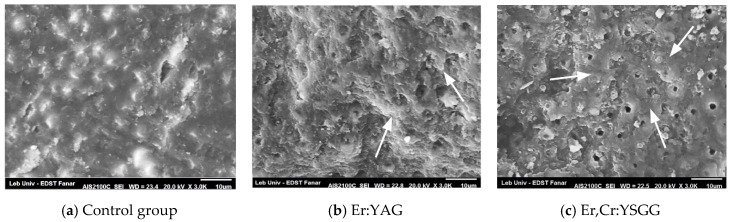
SEM observations of SBS test 24 h after bonding composite to deep dentin. (**a**) very little of resin left, absence of visible dentinal tubules probably due to the presence of smear layer. (**b**,**c**) Composite remnant in the intertubular dentin (arrows) with less intratubular infiltration.

Morphological assessment of the superficial dentin was observed under SEM for all specimens. Before thermocycling, the control group (Figure 2a) didn’t show any opened tubules, probably because of an existing smear layer over the dentinal surface. There was little remnant of resin left on the dentin. In group 2 and 3, dentin surfaces irradiated with Er:YAG and Er,Cr:YSGG revealed small numbers of opened tubule, as well as intertubular composite remnants (Figure 2b,c arrowed).

**Figure 3:**
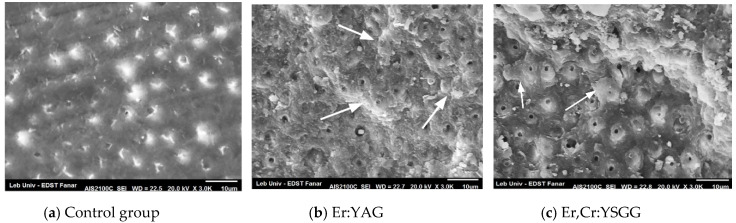
SEM views of thermal cycled specimens. (**a**) with very little opened tubules and absence of composite residue. (**b**,**c**) Few intertubular composite leftover (arrows).

After thermocycling, SEM observation of the fractured dentinal surfaces showed a clean dentinal surface in control Group 1 with almost no visible resin left over or infiltrating the tubules (Figure 3a). Composite remnants were present in both the Er:YAG and Er,Cr:YSGG groups, and more resin remnants were visible on the dentinal surfaces (arrows) than inside the dentinal tubules (Figure 3b,c). Group 2 and 3 showed less tubules closed with resin composite than dentin surfaces before thermal cycling.

**Figure 4:**
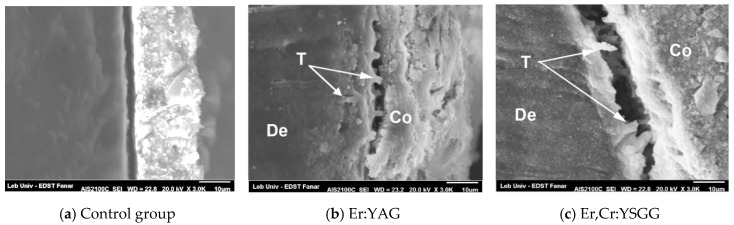
Longitudinal cut after thermocycling: (**a**) no tags were visible (**b**) and (**c**) visible tags with deep penetration of dentinal tubules (arrows). De: Dentin; Co: resin composite; T: tags.

Figure 4 represents a longitudinal cut of the laser-irradiated dentin after thermal cycling, specifically within the margins of the fractured zone. Resin tags penetrating dentinal tubules were clearly visible in Er:YAG and Er,Cr:YSGG specimens (Figure 4b,c) and absent in the control group (Figure 4a). A hybrid layer was too difficult to isolate due of the absence of bonding in SAC. 

## 4. Discussion

Although self-adhering flowable composites could be considered promising materials for restoring teeth with a simplified technique, their ability to bond on enamel and dentin was low [24,25]. In our study, mean SBS values were affected by laser-irradiation and thermocycling. Control group (Group 1) presented the lowest mean SBS value of 8.355 ± 2.297 MPa among all groups that were stored in water (WS). After thermocycling (TC), SBS of the control group dropped to a very low value while both laser-irradiated groups, Group 2 and 3, showed higher averages in SBS values with 13.10 ± 1.291 MPa and 14.04 ± 5.233 MPa, respectively, then dropped again after thermocycling but kept higher values than the control group. It was obvious that Er:YAG and Er,Cr:YSGG lasers enhanced mean SBS values. Significant differences were observed between the control group (Group 1) and both groups using lasers (Group 2 and 3) before thermocycling and preserved high SBS values after thermocycling. It may be possible that the low acidity of SAC stands behind the low SBS values causing fewer dentin tubules to be infiltrated [26]. Also, a correlation between acidity and the formation of a hybrid layer was demonstrated in a previous study [27]. Bonding with a mild acidic resin, similar to that of SAC (pH = 1.9), can slightly demineralize dentinal surfaces and expose the collagen fiber network resulting in a thin hybrid layer [27]. Additionally, weak SBS values of SAC bonded to dentin were reported when compared to standard bonding systems on permanent teeth [5,16,28,29] as well as on lacteal teeth [4]. The increase in SBS values after laser irradiations observed in our study may be explained by the fact that both lasers at a low level of energy could eliminate the smear layer and increase the contact of SACs’ acidic molecules with dentinal surfaces, allowing better collagen fiber network infiltration. A similar ascertainment was demonstrated in a previous study [29]. On the other hand, if a high level of laser energy is used, changes in morphological configuration of dentin and collagen fibrils would decrease bond strength to dentin [30]. 

The significant effect of thermocycling in decreasing the mean SBS of SAC has not been reported in the literature, while our study showed a significant difference of SBS values between nonlasered groups before and after thermocycling, as well as for Er:YAG and Er,Cr:YSGG groups. Similar results were reported by El-Araby et al. showing that after thermocycling a decrease in the shear bond strength of total-etch and self-etch bonding occurred to both enamel and dentin [31]. Huang et al. demonstrated similar results when using phosphoric acid and self-etch bond to dentin [32]. Bonding degradation was explained by Kawazu et al., stating that thermocycling would accelerate deterioration and initiate cracks of the resin/dentin interface by mean of thermal expansion [33].

### 4.1. Stereomicroscopic Observation

Based on our stereomicroscopic observation following SBS, both studied variables, laser- irradiation and thermocycling, exhibited no significant effect on failure mode. When SBS values dropped in TC-Group 1, ARI increased to score 5 where no composite leftover was observed. Moreover, the percentages of composite remnant were higher in laser irradiated groups. The latter may be explained by smear layer removal resulting in efficient impregnation of dentin with the adhesive agent [6], which may explain the high percentage of composite remnant on dentin that was maintained in laser irradiated groups (Group 2 and 3) after thermocycling when comparing failure mode with groups stored in water.

Resin infiltration into the collagen fiber network and dentin tubules is considered a major element for bonding success. Until today, Er:YAG and Er,Cr:YSGG use was not capable of substituting conventional bonding techniques. It was reported that no hybrid layer was observed after dentin irradiation with an Er:YAG laser when compared to SEA and multiple steps adhesives [34]. Moreover, Er:YAG and Er,Cr:YSGG lasers can alter the collagen fiber network configuration [30,35]. In our samples, Group 1 (control group) did not leave enough resin remnants on top of the observed dentinal surfaces after thermocycling due to weak acidity of SAC which prevented resin infiltration into dentinal tubules and hybrid layer formation. In both laser groups, specifically after thermocycling, leftover resin on dentinal surfaces presented lower percentage of failure than in the control group, probably due to infiltration of dentinal tubules rather than hybrid layer formation. Further studies may point out a significant lower percentage of failure.

### 4.2. SEM Observation

Our results showed that the Er:YAG laser, as well as the Er,Cr:YSGG laser, were able to eliminate the smear layer due to the infiltration of resin inside dentinal tubules Figure 1b,c and Figure 2b,c) while no significant resin infiltration was observed in the control group (Figure 1a and Figure 2a). This should explain the weak resistance of SACs against debonding if no laser irradiation was applied, especially after thermocycling. In a longitudinal cut, the presence of tags created by resin infiltration confirmed the expectation for the Er:YAG (Figure 3b) and Er,Cr:YSGG (Figure 3c) effects. Similar results predicted that an increase in the performance of SACs was due to the removal of the smear layer and the opening of dentinal tubules by laser irradiation [2,20], similar to when phosphoric acid was used [17,36], or Ethylenediaminetetraacetic acid (EDTA), which create conditions in which dentin inorganic components were supposed to dissolve [37]. This would explain the reason behind the significant differences in SBS obtained between the laser-irradiated and nonirradiated groups. On the other hand, the use of phosphoric acid would cause not only the removal of the smear layer but also the exposure of collagen fibers [38] thus creating a hybrid layer which has never been reported with laser irradiation.

A significant increase of laser energy could reduce the SBS values of bonding because it may damage, melt and denature the collagen layer and cause cracks and melting of hydroxyapatite in the dentin superficial layer, leading to its recrystallization [39,40]. In a previous study comparing laser multiple energy densities, low energy was proved to increase values of SBS of self-adhering flowable resin composite to dentin [20,29]. Even self-etch adhesive systems can’t perform the same way as multi-step systems regarding bonding strength and stability throughout time [41]. Previous studies have revealed the reasons behind weak adhesions, stating that self-etch adhesives (SEA) have high hydrophilicity and less hybridization potential [42]. Moreover, the quality of composite adhesion is mainly dependent on the quality of the hybrid layer and adequate infiltration between exposed collagen fibers [43]. It is supposed that the presence of a smear layer in the control group prevented SAC from infiltrating the dentinal microstructure and creating mechanical interlocking. Therefore, no composite remnants were left after the SBS test (Figure 2a and Figure 3a). The presence before thermocycling of composite remnants over tubules and intertubular dentin for the Er:YAG laser group (Figure 2b), and more specifically on intertubular dentin for the Er,Cr:YSGG laser group (Figure 2c) as well as after thermocycling (Figure 3b,c) can be explained by the fact that both lasers were able to enhance interlocking of SACs with dentin structure and opened tubules creating encrusted surfaces.. These results are in concordance with a study published by Taşar et al. [44].

### 4.3. Perspective

Based on our results, shear bond strength of self-adhering resin composite bonded to Er:YAG and Er,Cr:YSGG laser-irradiated dentin showed significantly higher values than nonirradiated dentin after 24 h, as well as after aging. Thus, the null hypothesis was rejected.

Previous studies revealed most of the advantages of using laser irradiation for bonding composite to tooth structure. Yet, enhancing bond strength and longevity of composite restorations can only be achieved when choosing the right combination of laser parameters. In this study, we demonstrated the advantage of using lasers at a lower energy density in order to eliminate the smear layer and enhance shear bond strength and longevity of self-adhering resin composite restorations.

Further studies might be useful to investigate the efficiency of low energy density on different types of bonding systems.

## 5. Conclusions

Within the limitation of our study it can be concluded that laser irradiation, among other techniques, whether using Er:YAG, or Er,Cr:YSGG lasers, improved self-adhering flowable composite (SAC) dentinal bond strength after thermocycling and significantly increased shear bond strength (SBS) in comparison to nonirradiated dentin.

## Figures and Tables

**Table 1:** Mean shear bond strengths (MPa) and standard deviations for the experimental groups with analytical results.

Storage Mode	Studied Groups	Mean ± SD	Std. Errors of Mean
**WS**	**Group 1**	8.355 ± 2.297 ^A^	0.6926
	**Group 2**	13.10 ± 1.291 ^B^	0.3727
	**Group 3**	14.04 ± 5.233 ^B^	1.511
**TC**	**Group 1**	2.75 ± 1.583 ^C^	0.4570
	**Group 2**	10.03 ± 1.503 ^A^	0.4339
	**Group 3**	10.53 ± 2.631 ^A^	0.7595

WS: Water Storage; TC: Thermal Cycled. Group 1 (control group) SAC (without irradiation). Group 2, dentin irradiated with Er:YAG. Group 3 dentin irradiated with Er,Cr:YSGG. Similar superscript letters indicate no significant difference between groups. Different superscript letters indicate statistically significant differences at *p* < 0.01.

**Table 2:** Frequencies and percentages of failure mode observed under stereomicroscope 20× magnification.

Studied Groups	Storage Mode	Failure Mode				
		Score 1 (%)	Score 2 (%)	Score 3 (%)	Score 4 (%)	Score 5 (%)
**Group 1**	**WS 24 h**	0 (0%)	0 (0%)	2 (12.16%)	4 (33.34%)	6 (54.5%)
	**TC**	0 (0%)	0 (0%)	0 (0%)	2 (16.67%)	10 (83.33%)
**Group 2**	**WS 24 h**	0 (0%)	0 (0%)	4 (32.19%)	5 (41.66%)	3 (26.15%)
	**TC**	0 (0%)	0 (0%)	2 (25.66%)	4 (32.34%)	6 (42%)
**Group 3**	**WS 24 h**	0 (0%)	0 (0%)	4 (33.84%)	5 (40.06%)	3 (26.10%)
	**TC**	0 (0%)	0 (0%)	3 (24.96%)	4 (31.34%)	5 (43.70%)
